# HMGB1 mediates splenomegaly and expansion of splenic CD11b+ Ly-6C^high^ inflammatory monocytes in murine sepsis survivors

**DOI:** 10.1111/joim.12104

**Published:** 2013-08-12

**Authors:** S I Valdés-Ferrer, M Rosas-Ballina, P S Olofsson, B Lu, M E Dancho, M Ochani, J H Li, J A Scheinerman, D A Katz, Y A Levine, L K Hudson, H Yang, V A Pavlov, J Roth, L Blanc, D J Antoine, S S Chavan, U Andersson, B Diamond, K J Tracey

**Affiliations:** 1 The Laboratory of Biomedical Sciences, The Feinstein Institute for Medical ResearchManhasset, NY, USA; 2 The Elmezzi Graduate School of Molecular MedicineManhasset, NY, USA; 3 Focal Area Infection Biology, Biozentrum, University of BaselBasel, Switzerland; 4 SetPoint Medical, Valen Inc.Valencia, CA, USA; 5 MRC Centre for Drug Safety Science, Molecular and Clinical Pharmacology, University of LiverpoolLiverpool, UK; 6 Department of Women’s and Children’s Health, Karolinska Institute and Karolinska University HospitalStockholm, Sweden

**Keywords:** anti-HMGB1, CD11b+ Ly-6C^high^, HMGB1, sepsis survivors, splenomegaly

## Abstract

**Background:**

More than 500,000 hospitalized patients survive severe sepsis annually in the USA. Recent epidemiological evidence, however, demonstrated that these survivors have significant morbidity and mortality, with 3-year fatality rates higher than 70%. To investigate the mechanisms underlying persistent functional impairment in sepsis survivors, here we developed a model to study severe sepsis survivors following cecal ligation and puncture (CLP).

**Methods:**

Sepsis was induced in mice by CLP and survivors were followed for twelve weeks. Spleen and blood were collected and analyzed at different time points post-sepsis.

**Results:**

We observed that sepsis survivors developed significant splenomegaly. Analysis of the splenic cellular compartments revealed a major expansion of the inflammatory CD11b+ Ly-6CHigh pool. Serum high-mobility group box 1 (HMGB1) levels in the sepsis surviving mice were significantly elevated for 4-6 weeks after post-sepsis, and administration of an anti-HMGB1 monoclonal antibody significantly attenuated splenomegaly as well as splenocyte priming. Administration of recombinant HMGB1 to naive mice induced similar splenomegaly, leukocytosis and splenocyte priming as observed in sepsis survivors. Interestingly analysis of circulating HMGB1 from sepsis survivors by mass spectroscopy demonstrated a stepwise increase of reduced form of HMGB1 (with known chemo-attractant properties) during the first 3 weeks, followed by disulphide form (with known inflammatory properties) 4-8 weeks after CLP.

**Discussion:**

Our results indicate that prolonged elevation of HMGB1 is a necessary and sufficient mediator of splenomegaly and splenocyte expansion, as well as splenocyte inflammatory priming in murine severe sepsis survivors.

## Introduction

Severe sepsis is a potentially lethal syndrome of organ dysfunction that occurs following infection or injury. There are more than 750 000 cases of severe sepsis annually in the USA, with an overall mortality rate of 20–30% 1,2. Recent epidemiological evidence indicates that survivors of severe sepsis are at significant risk of morbidity and death, with 3-year mortality rates higher than 70% 3. Severe sepsis survivors have significantly impaired quality of life and significant cognitive impairment and often require continuous support for activities of daily life 4–7. Despite the enormity of this major medical problem, and its high financial and human costs, the pathogenesis and mechanisms underlying the morbidity and mortality of ‘severe sepsis survivor syndrome’ are largely unknown.

We recently developed a murine model of severe sepsis survivor syndrome to investigate these issues 8. Animals were subjected to caecal ligation and puncture (CLP), which induces a lethal form of peritonitis associated with 30–60% mortality. Surviving animals were observed for 4 months; by all outward appearances, their behaviour appeared to be completely normal. However, sophisticated analysis of behaviour and memory revealed significant impairment of cognitive function. Serum levels of the pro-inflammatory cytokines, tumour necrosis factor (TNF), IL-1 and IL-6, were not increased significantly in the sepsis survivors, but significant increases in serum HMGB1 levels persisted for up to 6 weeks 8. Administration of neutralizing anti-HMGB1 monoclonal antibodies to sepsis survivors reversed the decline in cognitive function, implicating HMGB1 in the pathogenesis of severe sepsis survivor syndrome 8. Of note, it was previously shown in clinical studies that serum HMGB1 levels are significantly elevated in severe sepsis survivors at the time of hospital discharge following community-acquired pneumonia 9.

During the course of investigating this novel murine model of sepsis survivors, we unexpectedly observed significant splenomegaly in a time period that seemed to correlate with the appearance of delayed increases in serum HMGB1 levels. Here, we report that murine sepsis survivors develop persistent splenomegaly and leucocytosis with significant expansion of the inflammatory CD11b+ Ly6C^high^ monocyte subset in the splenic compartment. The kinetics of the onset of splenomegaly coincided with the appearance of C23–45 disulphide-linked HMGB1 (C23–45 HMGB1) and was attenuated by administration of anti-HMGB1 antibodies.

## Methods

### Mice

Adult male Balb/c mice weighing 20–25 g (Charles River, Wilmington, MA, USA) were used for CLP experiments. Animals were housed in standard conditions (room temperature 22 °C; 12-h light/dark cycle) and had free access to standard chow and water. Animals were allowed to acclimate for at least 8 days before experiments. All animal experiments were performed in accordance with the National Institutes of Health Guidelines under protocols approved by the Institutional Animal Care and Use Committee of the Feinstein Institute for Medical Research.

### Caecal ligation and puncture

Severe polymicrobial abdominal sepsis was induced by CLP as previously described 8. In brief, the caecum was isolated and ligated below the ileo-caecal valve and then punctured once with a 22-G needle. Approximately 1 mm of faeces was extruded, the caecum was returned to the abdominal cavity, and the wound was closed with surgical clips. One dose of antibiotic (imipenem/cilastatin 0.5 mg kg^−1^ diluted in a 0.9% saline solution) was administered immediately after CLP as a component of the resuscitation fluid (total volume of 1 mL). This model has an expected 50% mortality by day 7, with few or no deaths thereafter. In sham-operated animals, the caecum was exposed and then returned to the peritoneal cavity without further manipulation. Sham-operated control animals also received one dose of antibiotic treatment and resuscitation fluid as described above. At the *a priori* established weekly time-points, survivors were killed with CO_2_. Blood was collected by cardiac puncture and transferred to EDTA-coated tubes. Spleens were harvested in aseptic conditions and kept on ice until isolation of splenocytes.

### Splenocyte harvesting and *ex vivo* endotoxin challenge

Splenocytes were isolated using standard protocols and resuspended in red blood cell lysis buffer (5PRIME, Hamburg, Germany) for 10 min and then washed with phosphate-buffered saline (PBS). Cells were cultured in RPMI medium supplemented with 10% foetal calf serum, 100 U mL^−1^ penicillin and 100 μg mL^−1^ streptomycin (Gibco, Grand Island, NY). In a sterile, flat-bottomed 96-well plate, 2 × 10^5^ spleen cells were cultured for 24 h in 200 μL medium alone or in medium containing *E. coli*-derived endotoxin (25 ng mL^−1^; 0111.B4, Sigma-Aldrich, St. Louis, MO).

### *In vivo* treatment of sepsis survivors with the anti-HMGB1 monoclonal antibody 2G7

The anti-HMGB1 monoclonal antibody (mAb) 2G7 was generated as previously described 10,11. Mouse immunoglobulin (IgG)2b (ESMD Chemicals, Gibbstown, NJ, USA) was used as an isotype control. Sepsis survivors were injected with the anti-HMGB1 mAb 2G7 or isotype control IgG2b (50 μg per day intraperitoneally). Mice received one dose per day on days 9, 10 and 11 after the surgical procedure. Tissues were harvested for analysis on days 15 and 21 after surgery, that is, 4 or 10 days after the last dose of 2G7.

### Recombinant HMGB1 administration in healthy Balb/c mice

Recombinant rat HMGB1 was expressed in *E. coli* and purified as previously described 12,13. The HMGB1 preparation was tested for endotoxin (limulus assay was negative; data not shown) and activity (measured by *in vitro* induction of TNF production by RAW264.7 cells; data not shown). Recombinant HMGB1 (500 μg diluted in 350 μL PBS daily) or PBS alone was administered intraperitoneally to healthy Balb/c mice for 21 or 28 days. This dose of HMGB1 was found to induce an inflammatory response that lasts approximately 24 h 14. Blood was collected and the spleen was harvested 1 day after the last injection.

### Cytokine measurements

For plasma cytokine measurements, whole blood was collected by cardiac puncture using a 1-mL syringe containing 50 U heparin (APP Pharmaceuticals, Schamburg, IL, USA) and plasma was obtained by centrifugation at 240 ***g*** for 5 min. IL-2, IL-4, IL-6, IL-10, IL-17, interferon (IFN)-γ and TNF were measured by flow cytometry-assisted bead assays (BD Biosciences, San Jose, CA, USA) using a FACSArray instrument (BD Biosciences). IFN-α and IFN-β were measured by enzyme-linked immunosorbent assay (PBL Interferon Source, Piscataway, NJ, USA). Chemokine (C-X-C motif) ligand (CXCL-)1, interleukin (IL-)12p70, IFN-γ, IL-6, IL-10, TNF and IL-1β were measured using mouse pro-inflammatory 7-Plex kit (Meso Scale Discovery, Gaithersburg, MD, USA). HMGB1 levels were measured by immunoblotting analysis as described previously 12,13. Western blots were scanned with a silver image scanner (Silver-scanner II, Lacie Limited, Beaverton, OR, USA), and the relative band intensity was quantified using ImageJ software (v1.59, National Institutes of Health). Levels of HMGB1 were determined by reference to standard curves generated using purified HMGB1.

### Mass spectrometric characterization of the redox status of circulating HMGB1

For determination of redox modifications in sepsis survivors, HMGB1 was isolated by immunoprecipitation from plasma samples, as previously described 15,16. Proteins were then separated by nonreducing SDS-PAGE, and protein bands corresponding to the molecular weight of HMGB1 were excised and subjected to tryptic digestion. The resulting peptides were characterized by liquid chromatography and tandem mass spectrometry (LC-MS/MS) following differential alkylation as described by others 16. Individual peptide fragmentation to produce *b* and y ions was used to determine the amino acid sequence and confirm the presence of specific modifications. The relative abundance of various HMGB1 isoforms was assessed by determining the area under the curve using normalized extracted ion counts from the peptide peaks.

### Splenocyte characterization by flow cytometry

We characterized four cell groups: B cells (CD3^neg^, CD19^+^); T cells (CD3^+^, CD19^neg^); granulocytes (CD3^neg^, CD19^neg^, CD11b^+^, Ly-6G^+^); and monocytes (CD3^neg^, CD19^neg^, CD11b^+^, Ly-6G^neg^, Ly-6C^+^). Splenic and circulating monocytes were subdivided into *inflammatory* (CD11b^+^, Ly-6C^high^) or *resident* (CD11b^+^, Ly-6C^low^) cells as reported previously 17. Cells were stained for flow cytometry using the following antibodies: fluorescein isothiocyanate (FITC)-conjugated rat anti-mouse Ly-6C (eBioscience, San Diego, CA, USA); phycoerythrin (PE)-conjugated rat anti-mouse CD11b (BD Biosciences); PE-Cy7-conjugated rat anti-mouse Ly-6G (BD Biosciences); eFluor 450-conjugated rat anti-mouse CD3 (eBioscience); and allophycocyanin (APC)-conjugated rat anti-mouse CD19 (BD Biosciences). Intracellular staining was performed according to previously reported methods 18. Briefly, splenocytes were stained with FITC-conjugated rat anti-mouse Ly-6C, PE-conjugated rat anti-mouse CD11b and PE-Cy7-conjugated rat anti-mouse Ly-6G antibodies (as above). After washing, cells were permeabilized (with Cytofix/Cytoperm, BD Biosciences) and then stained with PE-labelled anti-mouse TNF, anti-mouse IL-6 or anti-mouse IL-10 antibodies (eBioscience). Data were acquired using an LSRII flow cytometer (BD Biosciences) and analysed with FlowJo version 9.0 (Tree Star, Inc, Ashland, OR).

### Statistical analysis

Data are expressed as mean ± SD. Differences between means were determined using two-tailed Student’s *t*-test. Survival analysis was performed using log-rank test. *P* values <0.05 were considered significant.

## Results

### Splenomegaly, weight loss and leucocytosis in severe sepsis survivors

**Figure 1:**
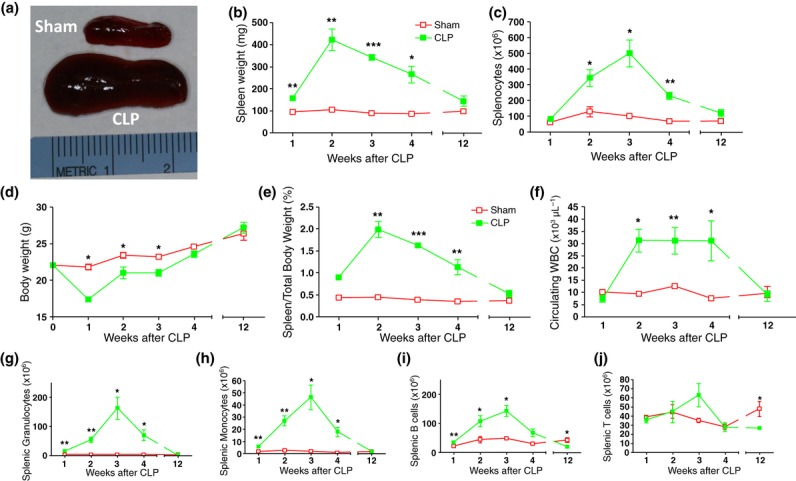
Sepsis-induced persistent splenomegaly, weight loss and leucocytosis. Balb/c mice were subjected to sham surgery or CLP. (a) Spleens from mice subjected to sham surgery or CLP 4 weeks after surgery. Spleen weight (b), total splenocyte count (c), body weight (d), ratio of spleen weight/body weight (e) and concentration of leucocytes in blood (f) in sepsis survivors and controls were measured at 1–12 weeks after surgery as indicated. (g–j), splenocytes were isolated from sham-operated mice or CLP survivors at different time-points after surgery and characterized by flow cytometry. The graphs show total numbers per spleen of granulocytes (g), monocytes (h), B cells (i) and T cells (j). Values are means ± SD (n = 5 mice/group). *P < 0.05, **P < 0.01 and ***P < 0.001 vs. control. WBC, white blood cell count.

Balb/c mice were subjected to CLP or sham surgery. The mortality rate was approximately 50% in the CLP group, and deaths primarily occurred during the first week; mice that survived for more than 7 days after CLP were included in the analysis. We observed splenomegaly and an increase in splenocyte number in the CLP group compared with sham-operated controls, for at least 4 weeks after CLP (Fig. 1a–c). Spleen weight peaked at 2 weeks after CLP and remained elevated at 4 weeks. As expected, total body mass was significantly reduced for the first 3 weeks in mice subjected to CLP compared with sham-operated control animals (Fig. 1d). This contributed to the significant differences in spleen weight/total body mass ratio observed in the experimental groups (Fig. 1e). Spleen enlargement in sepsis survivors was accompanied by significantly increased splenocyte counts (Fig. 1c) and persistent leucocytosis (Fig. 1f). These differences persisted for at least 4 weeks, and values normalized within 12 weeks after surgery.

### Splenomegaly is mediated by expansion of splenic granulocytes, monocytes and B cells

Splenocytes from murine sepsis survivors and sham-operated control animals were stained for cell surface markers and analysed by flow cytometry. In the first weeks after surgery, we observed a significant expansion of splenic granulocytes, monocyte and B cells in survivors that peaked 3 weeks after surgery. The largest cellular expansion was found in the granulocyte population (Fig. 1g), both in terms of absolute numbers (peak 162 ± 38 vs. 1.2 ± 0.4 × 10^6^ cells/spleen in sepsis survivors versus sham-operated controls) and fold increase (a 135-fold increase compared with sham-operated controls). Splenic monocyte counts were 23-fold higher (46 ± 10.0 vs. 2 ± 0.2 × 10^6^ cells/spleen; Fig. 1h) and splenic B-cell counts were threefold higher (143 ± 19 vs. 49 ± 4 × 10^6^ cells/spleen; Fig. 1i) in sepsis survivors compared with sham-operated controls. These responses are specific, as shown by the fact that splenic T cells failed to expand during this period (Fig. 1j). Within 12 weeks, the cell numbers within these subpopulations had returned to levels at or below those found in sham-operated control mice. Together, these results indicate that splenomegaly in severe sepsis survivors is associated with the expansion of granulocytes, monocytes and B-cell populations.

### Plasma C23–45 HMGB1 is significantly elevated for 8 weeks in severe sepsis survivors

**Figure 2:**
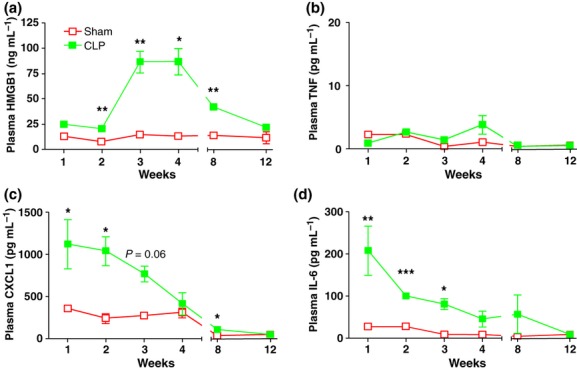
Cytokine levels after CLP. Balb/c mice were subjected to sham surgery or CLP. Levels of HMGB1 (a), TNF (b), IL-6 (c) and CXCL1 (d) were measured in plasma collected from control mice and murine sepsis survivors at 1–12 weeks after surgery as indicated. Values are mean ± SD (*n* = 5 mice/group). *P < 0.05, **P < 0.01 and ***P < 0.001.

Total serum HMGB1 levels, measured by quantitative immunoblotting, were significantly elevated for at least 8 weeks in severe sepsis survivors and returned to baseline levels within 12 weeks (Fig. 2a); this kinetic pattern correlates closely with the onset of splenomegaly. Plasma TNF, IL-6 and CXCL1 levels did not remain elevated during this period (Fig. 2b–d), indicating that the cytokine response is specific. Moreover, serum levels of IL-1β, IL-2, IL-4, IL-10, IL-17, IFN-α, IFN-β and IFN-γ were not elevated in severe sepsis survivors compared with sham-operated control mice (data not shown).

**Figure 3:**
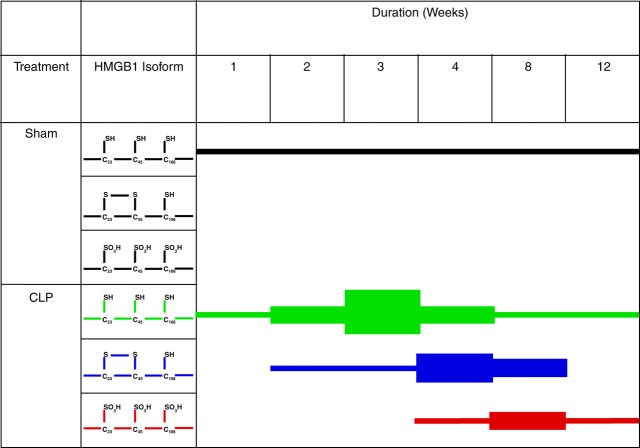
Mass spectrometric analysis of the redox status of the three cysteines (C23, C45 and C106) of HMGB1. Using LC-MS/MS, we demonstrated that HMGB1 follows a characteristic pattern: first the all-thiol form increases (peaking 3 weeks after CLP) followed by a rise in the disulphide isoform (predominant at weeks 4–8 after CLP), before the appearance of a terminally oxidized isoform lacking in inflammatory activity. The expression of each isoform at the relevant time is shown. The thickness of the horizontal black lines represents the relative magnitude of the each isoform at a particular time-point compared with other time-points.

Recent advances in understanding the molecular function–activity relationship of HMGB1 revealed that the redox status of the three cysteines of HMGB1 (C23, C45 and C106) critically influence its biological activities 15,19,20. Briefly, the all-thiol HMGB1 is abundant in the nucleus and is highly chemotactic; the C23–45 HMGB1 is pro-inflammatory; and the fully oxidized HMGB1 is biologically inert 15,19,20. To determine which form of HMGB1 is associated with the onset of splenomegaly in severe sepsis survivors, mass spectroscopy was used to assess the predominate isoforms of HMGB1 in murine plasma. We observed that circulating HMGB1 transitioned from predominantly the all-thiol HMGB1 during the second and third weeks after sepsis to the C23–45 HMGB1 during weeks 4–8. Finally, during weeks 8–12, the main form of HMGB1 was the oxidized, inert form (Fig. 3). Thus, accumulation of C23–45 HMGB1, which has been shown previously to be pro-inflammatory, occurs during the onset of splenomegaly in severe sepsis survivors.

### Expansion of Ly-6C^high^ splenic monocytes

**Figure 4:**
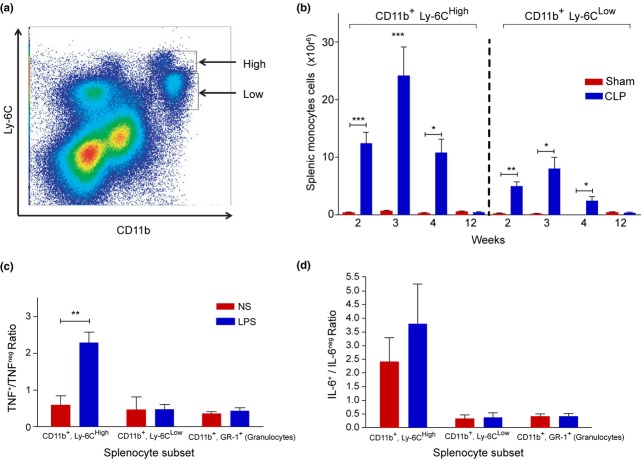
Sepsis survivors demonstrate a sustained expansion of inflammatory CD11b^+^, Ly-6C^high^, TNF-producing splenic monocytes. Splenocytes isolated form murine sepsis survivors or sham-operated mice at 2, 3, 4 or 12 weeks after surgery were stained for CD11b and Ly-6C and analysed by flow cytometry. (a) Gating for CD11b^+^ Ly-6C^high^ inflammatory monocytes and CD11b^+^ Ly-6C^low^ resident monocytes 4 weeks after sham surgery. (b) Numbers of inflammatory CD11b^+^ Ly-6C^high^ and resident CD11b^+^ Ly-6C^low^ monocytes per spleen in sham-operated or CLP-treated mice. Splenocytes were isolated from murine sepsis survivors at 4 weeks after surgery and stimulated with 25 ng mL^−1^ endotoxin in vitro for 60 min. After treatment, the proportion of TNF^+^ (c) or IL-6^+^ (d) splenocytes was determined by flow cytometry on the following cellular subsets: CD11b^+^ Ly-6C^high^ inflammatory monocytes; CD11b^+^ Ly-6C^low^ resident monocytes; and granulocytes. Data are shown as mean ± SD (n = 5 mice/group). *P < 0.05, **P < 0.01 and ***P < 0.001.

Recently, splenic monocytes have been functionally characterized as inflammatory (Ly-6C^high^) or resident (Ly-6C^low^) 17. To characterize these splenic cell populations, the surface expression levels of CD11b and Ly-6C were analysed by flow cytometry in sham-operated control mice and sepsis survivors (Fig. 4a). In control mice, the absolute numbers of Ly-6C^high^ and Ly-6C^low^ monocytes did not differ significantly between the investigated time-points after surgery. In sepsis survivors, both Ly-6C^high^ and Ly-6C^low^ monocyte subsets were significantly increased compared with control animals at 2, 3 and 4 weeks after CLP. The difference in absolute cell numbers between sepsis survivors and controls was much larger in the Ly-6C^high^ than in the Ly-6C^low^ population (Fig. 4b), indicating that splenic monocyte expansion is mainly due to inflammatory Ly-6C^high^ cells in sepsis survivors.

To further characterize the phenotype of these cells, splenocytes derived from mice 4 weeks after surgery were stimulated *ex vivo* with endotoxin for 60 min; intracellular TNF and IL-6 were analysed by flow cytometry. Endotoxin stimulation significantly increased the proportion of Ly-6C^high^ TNF^+^ inflammatory splenic monocytes, but did not alter the proportions of Ly-6C^low^ TNF^+^ resident monocytes or TNF^+^ granulocytes (Fig. 4c). No significant changes in proportions of IL-6^+^ cells were detected in any of the investigated cell subsets at this time-point (Fig. 4d), indicating that splenomegaly in severe sepsis survivors is associated with expansion of the TNF-producing Ly-6C^high^ monocytes.

### Administration of C23–45 HMGB1 mediates splenomegaly, leucocytosis and enhanced splenocyte response to endotoxin

**Figure 5:**
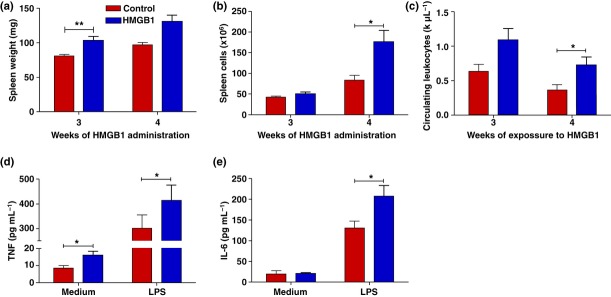
Prolonged administration of C23–45 HMGB1 in vivo reflects the septic immunophenotype. Balb/c mice received daily intraperitoneal injections of 500 μg recombinant HMGB1 or saline for 3 or 4 weeks and were then killed. The immunophenotype was compared between HMGB1- and saline-treated animals: spleen weight (a), splenocyte count (b) and circulating white blood cell (WBC) counts (c) were measured. Splenocytes were isolated and cultured for 24 h with or without 25 ng mL^−1^ endotoxin. TNF (d) and IL-6 (e) in the culture medium were measured by cytokine bead array. Values are mean ± SD (n = 5 mice/group). *P < 0.05 and **P < 0.01.

To study the role of elevated HMGB1 levels in this model, healthy mice received daily administration (500 μg per day intraperitoneally) of recombinant C23–45 HMGB1 (C23–45 rHMGB1) for up to 4 weeks. Animals exposed to C23–45 rHMGB1 developed splenomegaly and increased splenocyte counts (Fig. 5a,b) as well as leucocytosis (Fig. 5c). Stimulation of splenocytes with endotoxin *ex vivo* resulted in significantly increased levels of TNF and IL-6 compared with splenocytes isolated from control animals (Fig. 5d,e). Together, these data indicate that chronic exposure to HMGB1 is sufficient to mediate splenomegaly and leucocytosis *in vivo* and enhance cytokine responses to endotoxin in splenocytes *in vitro*.

**Figure 6:**
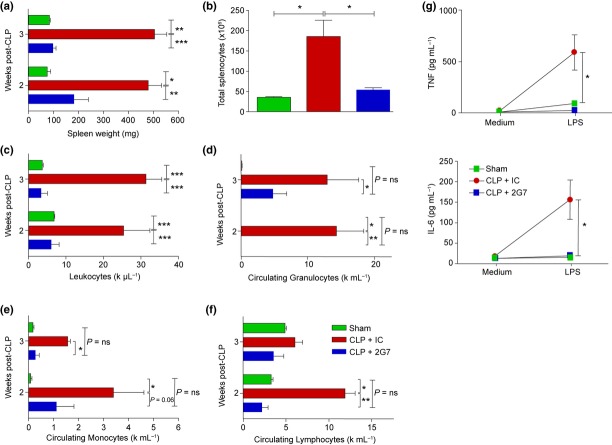
Administration of the neutralizing anti-HMGB1 mAb 2G7 in murine CLP survivors reverses splenomegaly, leucocytosis and splenocyte priming. Murine sepsis survivors were randomly assigned to receive 50 μg per day of either neutralizing anti-HMGB1 mAb (2G7) or isotype control (IC) on days 9–11 after CLP. Whole blood and spleens were collected on days 15 or 21 after CLP (4 and 10 days after the last dose of 2G7). Spleen weight (a), total splenocyte count (b) and circulating leucocyte (c), granulocyte (d), monocyte (e) and lymphocyte (f) counts were measured (n = 4–5 mice/group). (g) Splenocytes were isolated on day 21 after surgery from sham-operated animals or CLP survivors that had been treated with 2G7 or PBS; 2 × 10^5^ cells/well were challenged with 25 ng mL^−1^ endotoxin for 24 h ex vivo. TNF and IL-6 levels in the culture medium were measured by cytokine bead array. Data shown are means ± SD (n = 5 mice/group). *P < 0.05, **P < 0.01 and ***P < 0.001.

Next, we administered the anti-HMGB1 mAb 2G7 (shown previously to neutralize C23–45 HMGB1) to severe sepsis survivors once daily on days 9, 10 and 11 after surgery. These selected time-points were likely to be after all expected mortality events due to the acute illness but prior to the onset of peak HMGB1 levels. Administration of 2G7 significantly protected against the development of splenomegaly (Fig. 6a) and splenocytosis (Fig. 6b). Splenocyte counts in the 2G7-treated mice were similar to those of sham-operated control mice. The anti-HMGB1 mAb also attenuated leucocytosis, with significant decreases observed in circulating granulocytes, lymphocytes and monocytes (Fig. 6c–f). The anti-HMGB1 mAb also significantly attenuated TNF and IL-6 release from endotoxin-stimulated splenocytes *ex vivo* (Fig. 6g). Thus, together with previous results, our findings suggest that C23–45 HMGB1 is a necessary and sufficient mediator of splenomegaly, leucocytosis and enhanced endotoxin responses in splenocytes in murine sepsis survivor syndrome.

## Discussion

Splenomegaly was described as an important complication of acute and chronic infections more than 100 years ago 21–23. The findings of the present study indicate that the pathophysiology of this phenomenon in sepsis survivors may be dependent upon HMGB1. Furthermore, our finding that exogenous administration of C23–45 rHMGB1 in healthy mice is sufficient to cause expansion and increased cytokine response of splenocytes suggests that elevation of HMGB1 can mediate the long-lasting changes in immunophenotype observed in murine sepsis survivors. Previously, we showed that administration of anti-HMGB1 2G7 significantly attenuated the cognitive dysfunction in murine severe sepsis survivor syndrome 8. Here, 3 days of treatment with the anti-HMGB1 mAb 2G7 in sepsis survivors normalized splenocyte counts and numbers of circulating neutrophils, lymphocytes and monocytes, as well as the cytokine response to endotoxin, indicating that HMGB1 is necessary for the development of this immunophenotype in murine sepsis survivors. A range of diseases with inflammatory components are over-represented in human sepsis survivors 24, and inflammation has been suggested to underlie the excess morbidity and mortality in these patients 25. It is interesting that individuals who survive sepsis also demonstrate elevated levels of HMGB1 9,26. Pharmacological agents directed against HMGB1 are currently under clinical development, and it will be extremely interesting to explore their efficacy in preventing the complications of severe sepsis survivor syndrome.

In murine endotoxaemia, the spleen is the main organ source of systemic TNF, and splenic monocytes/macrophages are the major TNF producers 27. Amongst splenic monocytes, the Ly-6C^high^ inflammatory type showed the most pronounced increase in absolute numbers, and this population responded with the strongest increase in TNF in response to inflammatory stimuli. Our observation that C23–45 HMGB1 stimulates splenocytes to produce more TNF in response to endotoxin *ex vivo* suggests that serum HMGB1 elevation in murine sepsis survivors can contribute to the observed monocyte population shift towards the Ly-6C^high^ TNF production-prone phenotype. The expansion of splenic leucocytes we observed in murine sepsis survivors does not account fully for the increase in total spleen weight, and it is likely that other factors such as expansion of red pulp, stress haematopoiesis or fluid redistribution also contribute to the splenomegaly in sepsis survivors.

In conclusion, the present findings indicate that elevated serum C23–45 HMGB1 in murine severe sepsis survivors mediates splenomegaly and leucocytosis with expansion of the Ly6C^high^ monocyte subset and primes splenocytes to endotoxin stimulation. An anti-HMGB1 mAb, which has previously been shown to attenuate cognitive dysfunction in murine severe sepsis survivors, also attenuates the immunophenotype and splenomegaly.
